# Exploring Psychological Constructs in People Receiving Treatment for Addictive Eating Behaviours: “I Hate Loving Food as Much as I Do”

**DOI:** 10.3390/bs13100817

**Published:** 2023-10-04

**Authors:** Rebecca A. Collins, Kerith Duncanson, Janelle A. Skinner, Phillipa J. Hay, Susan J. Paxton, Tracy L. Burrows

**Affiliations:** 1 School of Health Sciences, The University of Newcastle, Callaghan, NSW 2308, Australia; janelle.skinner@newcastle.edu.au (J.A.S.); tracy.burrows@newcastle.edu.au (T.L.B.); 2 Food & Nutrition Program, New Lambton, NSW 2305, Australia; kerith.duncanson@newcastle.edu.au; 3 School of Medicine and Public Health, The University of Newcastle, Callaghan, NSW 2308, Australia; 4 Translational Health Research Institute, School of Medicine, Western Sydney University, Sydney, NSW 2560, Australia; p.hay@westernsydney.edu.au; 5 Campbelltown Hospital, South West Sydney Local Health District, Sydney, NSW 2560, Australia; 6 School of Psychology and Public Health, La Trobe University, Melbourne, VIC 3086, Australia; susan.paxton@latrobe.edu.au

**Keywords:** addictive eating, compulsion, craving, control, normal

## Abstract

Research into the complexities of addictive eating behaviours continues to develop, as a deeper understanding of this construct beyond self-report diagnostic tools emerges. In this study, we undertook structured interviews with 40 participants engaged in a personality-based management program for addictive eating, to gain insight into what situations lead people with addictive eating behaviours to overeat, and how they believe their lives would be different if they had control over their eating. A phenomenological analysis to explore compulsion and control in the context of food experiences for participants was used to construct two main themes of the addictive eating paradox and striving to transition from ‘other’ to ‘normal’. The addictive eating paradox identified multiple contradictory experiences of a situation, e.g., ‘loving food’ but ‘hating food’. *Striving to transition from ‘other’ to ‘normal’* encompassed the idea that participants envisaged that by gaining control over their eating they could become ‘normal’. This study emphasises the need to provide support and strategies to help people navigate paradoxical thoughts and presents new ideas to increase the effectiveness of interventions for individuals struggling with the complex self-beliefs held by those with addictive eating behaviours.

## 1. Introduction

Addictive eating continues to gain substantial research attention, thus contributing to the ongoing exploration into the complex relationship between humans, food and eating behaviours. Although not currently recognised by the Diagnostic Statistical Manual of Mental Disorders 5 (DSM-5-TR) [1], addictive eating can be characterised by a distinct pattern of persistent, recurrent, often excessive, compulsive, and uncontrolled food consumption [2,3]. The Yale Food Addiction Scale (YFAS), the most commonly used self-report tool for addictive eating, is a valid scale that supports the construct of addictive-type eating and allows addictive-type eating to be conceptualised in the same way as other substance use disorders using DSM-5-TR diagnostic criteria [1,2]. However, it is important to note that the construct of addictive eating is still a highly debated and controversial area of research [4,5,6]. Currently, there are minimal evidence-based behaviour management programs available for people with self-reported addictive eating, with majority of interventions being weight loss-focused and based around bariatric surgery, medications, and lifestyle interventions [7]. To assist in the development of effective management programs, there is a need for the experiences and perspectives of those with addictive eating behaviours to be understood and for further clarity on the individualistic nature of addictive eating behaviours to be identified.

Addictive eating is highly variable, with differences in individual experiences being reported [8,9,10,11]. It is found in diverse mental health disorders including anxiety, depression, post-traumatic stress disorder, attention deficit hyperactivity disorder, and eating disorders including binge eating disorder (BED) and anorexia nervosa (AN) [12,13]. Qualitative research allows further exploration of the non-homogenous aspects of the behaviour to complement and add to the existing quantitative research and self-report diagnostic tools. A recent qualitative evaluation study of the Yale Food Addiction Scale 2.0 (YFAS), the most commonly used self-report tool for addictive eating, highlighted the reliability of the YFAS 2.0 to capture the experience of addictive eating. However, new themes were identified including; emotional eating/eating to cope, secretive eating, and weight gain [14]. The connection between addictive eating and weight gain has been explored in previous studies as a stressor and a driver of the desire for control over eating behaviours [8]. Further understanding of these novel themes will aid in the progress of addictive eating research and ensure that what is being captured in diagnostic tools is being incorporated into constructive behavioural and lifestyle management programs.

As compulsion and control consistently arise in the investigation of the experiences of those with addictive eating, it is important to gain further insight as to the full meaning of these concepts to those with addictive eating behaviours. This allows adults with addictive eating to provide their interpretation of these topics and the connection of compulsion and control to their personal stories of eating [8,10]. Compulsion in the context of addictive eating behaviours is often viewed as a driving factor hindering control over one’s eating behaviours [8]. Compulsion of any description can leave individuals feeling disconnected from their personal goals, by continuing to engage in behaviours despite negative personal or health outcomes [15]. Compulsion in addictive eating appears to manifest predominantly as the YFAS clinical impairment symptom of craving, whereby individuals are unable to regulate food consumption when presented with related cues (e.g., visual or situational) [2,8,16,17]. Control has strong associations with addictive and substance use disorders, and participants in qualitative addictive eating studies frequently discuss their experiences with a lack of control or desiring control [8,9,11]. The idea of control in addictive eating goes beyond a mere lack of control over eating behaviours, but becomes an essential aspect of individual efforts to manage food intake [8]. Research has documented that rigid control in dieting behaviours may lead to increased cravings and a perpetuation of the addictive eating cycle and societal pressures around how one should eat and perceptions of body weight may also play a driving role in the desire for control in individuals with addictive eating behaviours [8,18].

Thus, the aim of this study was to gain insight into the perceptions of compulsion and control of Australian adults with addictive eating behaviours in a qualitative study which addressed two questions: (1) what situations lead people with addictive eating to overeat, and (2) how do people with addictive eating behaviours believe their lives would be different if they had control over their eating?

## 2. Method

### 2.1. Research Design

To gain an understanding of perceptions of control and drivers of overeating among adults with addictive eating behaviours, brief qualitative interviews and the methodology of phenomenology were adopted [19]. Phenomenology was used to facilitate exploration of the phenomena of compulsion and control in the context of food experiences for participants and was appropriate to contextualise how these experiences play out in day-to-day situations.

### 2.2. Recruitment and Sampling

Participants were engaged in a personality-based management program for addictive eating, known as the TRACE program (Targeted Research in Addictive and Compulsive Eating) [20]. Participants were recruited via media releases, local and national advertising, and social media. The TRACE program was implemented as a randomised controlled trial with three parallel arms (*n* = 58, arm 1; *n* = 60, arm 2; *n* = 57, arm 3) [20]. Recruitment ran from August 2021 until April 2022. To be included in the study, participants had to be between the ages of 18–85 years, reside in Australia, endorse at least three symptoms on the YFAS 2.0 [2] (a validated 35-question tool with participant responses summed to obtain a total symptom score ranging from 0–11), have a self-reported weight and height consistent with a body mass index (BMI) of ≥18.5 kg/m^2^, be proficient in English, and have access to the internet. Individuals who were pregnant or lactating, reported severe mental illness (e.g., schizophrenia or bipolar disorder), or reported purging behaviours identified using the Eating Disorder Examination Questionnaire—Short form EDE-QS [21] were excluded from the study.

Participants were invited to this qualitative sub-study if they had been recruited to arm 1 (active intervention group) of the TRACE study. These individuals participated in five telehealth sessions with an Accredited Practising Dietitian, received a participant program workbook, and had access to the TRACE website that contained supporting resources. The questions for the current study were asked at the beginning of telehealth session one.

### 2.3. Measures

Participants were required to complete a baseline eligibility questionnaire. Refer to TRACE program protocol for full details [20]. Demographic details including; age, sex, self-reported height and weight (which was later converted to BMI), education, relationship status, employment, and living situation were collected.

Data gathering for the current study was via a brief, structured interview at the beginning of the first telehealth session of the TRACE program. The questions for the interviews were developed and verified for relevance with an experienced qualitative researcher. The questions were based on the themes from previous investigations into the experience of food addiction in Australian adults [8]. The two interviewers were Accredited Practising Dietitians, with more than six years one-on-one dietetic counselling experience. The interviewers discussed the responses being received from the participants to the questions and deemed the two questions appropriate to gain accurate and rich data on the phenomena of compulsion and control.

Participants were asked two standardised questions. As the interviews were opportunistic within the TRACE program, follow-up questions were not asked of the participants.

1. What situations most often lead you to overeating? Can you describe a recent example of one of those situations?
2. How would your life be different if you had control over your eating?

### 2.4. Procedures

This study was approved by the University of Newcastle Human Research Ethics Committee (H-2021-0100) and was prospectively registered with the Australian New Zealand Clinical Trials Registry (ACTRN12621001079831). Informed consent was obtained for both questionnaires and recording of the interviews prior to randomisation.

The interviews were audio-recorded by the session facilitator and transcribed for analysis using a transcription service (Outscribe https://www.outscribetranscription.com.au) (Accessed on 20 February 2022). Each transcript was manually checked to ensure accuracy and completeness. All identifying or potentially identifying information was removed from the transcripts to ensure anonymity of participants. Identification numbers were assigned upon entry into the TRACE Program (participant numbers range between 008 and 716) and were used in place of participant names for the current study.

### 2.5. Data Analysis

Data were analysed independently by two researchers (RAC and KD) in the QSR NVivo 12 [22] qualitative analysis program using the procedures for reflexive thematic analysis as outlined by Braun and Clarke (2006) [19]. For the current analysis, the following process was used:

Familiarisation with data: Transcripts were read and re-read, noting initial impressions relating to the study aims. Reviewers met to discuss overall first impressions of the transcripts and to discuss notable or interesting descriptions discussed by the participants.

Coding data: Transcripts were coded in NVivo separately by each reviewer. Transcripts were examined line by line, and codes assigned to begin to characterise the data. Initial codes were organised into categories by grouping similar codes together. Multiple meetings were held to discuss codes that had been created, to discuss similarities, and how both researchers viewed how these codes were describing the data. These discussions resulted in the generation of the final coding framework through a combination of codes from both researchers.

Transforming codes into themes: Preliminary themes were identified and began to describe how the data were relevant within these themes.

Synthesising themes: Themes from both researchers were similar, and discussions were had regarding interpretation and prominence of themes, potential overlap between themes, and checking that the themes being described were backed up by the data in the participant transcripts.

Defining and naming themes: The themes were grouped and then named with relevant sub-themes included. Themes were then discussed again, and then taken to the wider research group to ensure all data was represented and that the themes could be clearly explained and understood.

### 2.6. Reflexivity

RAC has been working with adults with addictive eating for six years and had undertaken the previous qualitative study of adults’ experiences with addictive eating. KD has experience working with eating disorders and qualitative research and has a thorough understanding of eating behaviours. Reflexive journals were kept during the coding process, with multiple discussions occurring between the two authors to ensure clear logic between the data contained in the transcripts and the codes and themes constructed. Where divergent interpretations of data were reported, perspectives were discussed and, in some instances, both interpretations are described, as per Braun and Clarke description of reflexive thematic analysis [19].

## 3. Results

### 3.1. Participants

Fifty-eight participants were randomized to participate in the active intervention arm of the TRACE study with forty participants completing session one. All forty participants completed the interview stage of session one. All forty recordings were able to be accurately transcribed and included for analysis. Participant demographics are outlined in Table 1.

### 3.2. Themes

The data from participant responses to questions about situations leading to overeating and control were characterised by discord within the participants, producing two predominant themes (Table 2): The paradox of addictive eating, and improvement of Oneself.

### 3.3. Theme 1: The Addictive Eating Paradox

The outcomes participants sought from being in control of their eating contrasted with the situations that led them to overeat. Many participants appear to be in a “chicken or the egg” state, which perpetuated an infinite loop of persistent addictive eating behaviours. The theme of the addictive eating paradox included three subthemes. The love of food subtheme, which describes the strong positive descriptions participants provide about food coupled with a strong hatred of their love of food. The drivers of overeating subtheme characterised participant eating behaviour because the drivers of overeating tended to be propelled, rather than solved, by the overeating. The boredom and busyness dichotomy subtheme describes how participants eat due to boredom but opposingly describe the burden of being busy driving them to eat.

#### 3.3.1. The Love of Food

The participants’ love of food was a frequent talking point throughout their interviews. Some participants expressed love for trying all foods due to the fear of missing out, eating out, cooking meals, and also reported spending an inordinate amount of time thinking about foods. Yet, this love of food came with negative statements about hatred towards food. Few participants described looking forward to meals with anticipation. Participants expressed regret for the time spent worrying about food and what they ate and, therefore, not enjoying food-related activities.

Participant 212 described the experience of missing out:

*“If there’s a lot of delicious food, I would like to try a bit of everything. I love food. I love colour, love different cultural foods. Someone who finds it hard to choose, if you gave me a box of chocolates, I would want to try each flavour, or one of those boxes that had the little jellybeans of multi-flavours or something, it’s that FoMO of “what am I missing out on? Let’s say I had that one and that one was better. Maybe I should try one of those”. So, it’s that satiation of the tastebuds”.*

Participant 716 described a similar thought pattern:

*“Fear of missing out on something new, something interesting, new flavours, new tastes. That’s on special, that’s half price, that’s marked down. So that fear of missing out”.*

Participant 553 discussed cooking for the family and eating repeated meals:

*“…then I’ll feel like I’m missing out on any other food. So, I’ll start eating normally, plus eat an extra two or three servings from the meal kit”.*

Along with this came a preoccupation with thinking about food, with participant 127 stating that:

*“Food is something I think about from the minute I wake up to the minute I go to sleep”.*

This love of food and the amount of time spent thinking about food would then be expressed by participants as something that was consuming their time and inhibiting their overall quality of life.

Participant 52 asserted that they were:

“*…actually just sick of it dominating so much of what I think about*”,

and participant 242 felt like:

“*I’d actually get jobs done” and “…I could focus on thinking about real-life things and not just thinking about food all the time, which I feel like I do, which is pretty sad*”.

This idea was reinforced by participant 475:

“*it takes up a lot of time, thinking and planning my day around when I’ll eat and what I eat. So, I would have a little bit more time to think about other things and do other things*”.

The majority of these expressed thoughts by the participants described a strong appreciation of food, coupled with the regret their preoccupation and desire for food has caused. This notion is succinctly summed up by participant 418, who states:

“*I love food, and I hate that I love food*”.

The participants expressed their deep love of food coupled with associated negative feelings and regret. Despite their high levels of appreciation for food, a common sentiment was a combination of both, love and frustration, when it came to food and eating.

#### 3.3.2. Drivers of Overeating

Overall, the participants described their belief that being in control of their eating would lead to a reduced level of stress and/or anxiety. However, stress and anxiety were some of the main drivers of overeating for participants. Participant 127 felt that:

“*…if I was able to control the thoughts along with that overeating, I would just be a lot less stressed. A lot calmer*”.

Conversely, the same participant stated later in their interview that what drives them to overeat is:

“*…working from home with the pantry right there, boredom and stress…I find that probably stress is the biggest part for me when I get stressed and it could be something so minor. I will just go straight for food for comfort*”.

This incongruity highlights another paradoxical aspect of addictive eating; I eat because I’m stressed, but I’m stressed because I eat. Participant 259 describes:

“*the perfect storm is when I’m really tired, I’m really stressed, and I’m feeling really down about myself*”.

These drivers were commonly expressed by participants and further emphasised the discord between the achievement of control of eating and the drivers of their addictive eating behaviours. Many participants reported intentional use of food as a reward. Participant 52 referred to using food as a reward multiple times:

“*because you’re doing this, well’’ reward you’. So you can have chips or you can have some chocolate or you can have some diet coke and whatever you need to get through*”.

Participant 328 reiterated this point, simply stating:

“*So, I see food as ‘I’ve done something good, so therefore, I deserve this*”.

Similarly, participants sometimes had the intention to use food to ‘feel better’ to offset negative thoughts or recover from an event that occurred in their day. They reported that eating food is their default reaction to whatever had caused them to feel overwhelmed. Participant 140 describes this type of occurrence:

“*I do get a lot of anxiety around a lot of different situations and I find, especially being a stay-at-home mum with the two kids now, and working from home, COVID and all the rest of the fun stuff, I find that the higher my anxiety levels, the more I turn to food that I feel, like sugary foods, chocolates, that will make me, in my head, calm me down, make me feel better for that instant and help reduce the anxiety.*”

Despite this repeated behaviour not leading to a positive outcome, participants do not change the behaviour:

Participant 52 ended their reflection of eating as a reward by saying:

“*So, then you spend the whole day eating crap and probably going high and low with the sugar high and then the flat, well, then I need something else*”.

This paradox of eating, being both causal and inducing effect factors of stress and anxiety for participants, highlights the complex relationship between mental health and eating behaviours. Also highlighted is the paradox of using food to alleviate negative affect, despite positive effect rarely being reached.

#### 3.3.3. The Boredom and Busyness Dichotomy

Boredom was commonly discussed by participants as a driver for overeating. Interestingly, it became apparent within the responses that many participants experienced boredom eating but would also overeat when they felt overwhelmed with too many tasks to undertake—the opposite of boredom. Participant 185 described this as:

“*…a tendency to eat when I’m bored or when I’m trying to relax*”

Followed by a subsequent comment that their overeating was:

“*…mostly to do with the feeling of being overwhelmed or being really stressed*”.

Participants expressed that one of the best strategies to avoid overeating was to keep busy and avoid boredom, yet both boredom and busyness triggered searching for and consuming foods. Participant 547 talked about eating when bored:

“*…just boredom. I find if I’m busy is the best thing, because I don’t think about food as much, and just keeps me preoccupied. And then if I’m bored, doing something on the computer or whatever, then that encourages me to eat poorly*”.

Many participants engaged in furtive eating when they did not want others to see them eating and related this to boredom. Participant 547 describes boredom as a driver for overeating, yet seeks time away from people to be alone and eat the foods they desire,

“*being on my own, or boredom. So yeah, being on my own. So, I tend to eat the bad food, like, if I pick up X or chips or whatever, I tend to do that on my own. And I’m married here, but I wouldn’t sort of bring X into the house and eat that, say, in front of my husband or, you know, my family. I would do it when they weren’t here or if I was out and about.*”

Keeping busy was viewed as a strategy by participants to avoid eating. However, participants acknowledged that both boredom and busyness prompted their search for and consumption of food. The effects of these two opposing states underscores the complex nature of addictive eating behaviours.

### 3.4. Theme 2: Striving to Transition from ‘Other’ to ‘Normal’

Participants discussed their belief that they expected gaining control of their eating would improve who they were as a person. Participants engaged with ‘othering’ by labelling themselves as different from what they perceived to be the social norm. This included both physical and mental aspects of self. The theme of striving to transition from ‘other’ to ‘normal’ has two subthemes: *Mending psychological and health impacts and Desiring normality. Mending psychological and health impacts* encompasses participants predicting they would feel happier with themselves if they were in control of their eating. This subtheme was additionally characterised by the desire to control food intake and thereby control aspects of their health, including weight and chronic health conditions (e.g., diabetes and blood pressure). *Desiring normality* incorporated striving to fit in with social norms and perceptions of other people and what they view as normal eating behaviours.

#### 3.4.1. Mending Psychological and Health Impacts

Participants discussed happiness as an aspirational goal of being in control of their eating. They connect control with happiness regarding their feelings towards themselves. Participant 547 describes this as:

“*I would hope that I would feel happier in myself because whilst I enjoy the crap food I am having at the time, I feel ashamed of myself afterwards for having had that crap food, and just it’s a vicious circle then. It just makes you feel crap, and then you eat more rubbish, and so on and so forth. So, I would hope that I would feel less ashamed, and I’d feel better about myself in general.*”

Participant 97 simply stated:

“*I think I’d be happier and healthier because I’m not so negative towards myself, I suppose*”.

Happiness and healthfulness were often connected in the participants’ minds, describing improvements in health conditions and weight status as ways to achieve happiness. However, participants described many complexities around this achievement of happiness. Participant 334 expressed:

“*I would be healthier, i.e., weight loss, things that I need to take tablets for, blood pressure, cholesterol. I’ve got osteoarthritis so if I lose weight, if I get on top of this eating business, I’d be more mobile. I would like my body better…I could wear clothes that I really like, but don’t fit. I think I’d be happier because I would like myself more and then, I would be less afraid of being judged.*”

Participant 385 also links their happiness to health:

“*…I think I would feel happier. I wouldn’t be as critical of body image and things along those lines. I would feel healthier because I think I would be able to lose weight over time and as I’ve put on more and more weight, over the past couple of years, for a multitude of reasons, I don’t feel as healthy as I used to, and I know how important it is. I want to get there, but it’s just been a challenge. I think also it’s not super healthy, not only do I think about food, I constantly think about health, eating, and body image all intertwined in quite a critical way, which probably from a mental standpoint is more likely to lead to something like a depression down the track or just unhealthy throughs, as well as the unhealthy behaviour.*”

Control was viewed by participants as a path to happiness. Control was coupled with positive emotions and improved health. Happiness was strongly connected to improvements in health conditions, weight loss and self-acceptance. However, it was also acknowledged that challenges such as body image, preoccupation with food, and self-criticism were barriers to achieving this control, and therefore, being able to achieve health and happiness.

#### 3.4.2. Desiring Normality

Desiring normality was described in different ways by participants. Some described normality as just being able to feel normal, which was sometimes articulated as “fitting in”. The participants’ ideas around normality were evident when expressing their thoughts around control of eating. Participant 164 voiced their idea of normal:

“*I mean, I would love to be what I would call ‘just normal’, be able to have a small meal.*”

Participant 328 described their perception of normal in more depth and experiences of having their “abnormality” confirmed:

“*…I’ve gone down so many different avenues and I just cannot, it frustrates me so much that I cannot solve the problem. I just think ‘I wish I was normal’ and some people will, which don’t have the same issues, also reflect that back to me when they would say things like ‘I don’t understand, stop eating or just exercise more or control your food intake’ and I think ‘yeah, I wish I could do that and you can, so you’re normal and I’m not.*”

This concept of normality was coupled with worry about other people’s perceptions of participants, and their lack of control over their eating. Participants would adjust their eating behaviours to appear ‘normal’ and fit in. Participant 463 stated:

“*I never eat out of control when I’m with other people. Like, I’m a very controlled eater when other people see me eat.*”

Participant 574 describes the pre-emptive steps taken to accomplish fitting-in:

“*Also, I worry about the amount of food I eat around people, so that also restricts, like when I agree to go to places, I’ll always think, I’ll look at menus, I’ll think this is what I’m going to order, but then that doesn’t always work out. It’s just worries about my consumption and how I eat around people as well.*”

Others voiced their desire for normality through the observation of others and what they perceived to be normal eating behaviour. Participant 164 commented:

“*I often look at people that seem to be in control of what they do, like of how they eat and what they eat, and I feel really envious of them being able to do that.*”

Participant 198 described their observation of others in social eating situations:

“*And I’m always ashamed if I eat a larger meal than other people or I have eating habits that inhale my food, rather than slowly eating it at a pace that my friends might be eating.*”

Normality was viewed by participants as being able to control their meal sizes and not having to face constant challenges with their eating. They expressed wanting to fit in with others and to feel like they belong in social situations. Participants were concerned with others’ perceptions of them, and their eating and body and could feel envious observing those that they perceived as “normal”.

#### 3.4.3. Social Confidence

The idea of being more confident in a social setting appeared to be an important factor in gaining control of their eating for participants. Participant 23 explained:

“*I would be more confident to join in with certain things, to do certain things like dancing, or where people are looking at me, I don’t participate if I’m going to be a focus…*”

Participant 484 considered what they might be like once eating control had been obtained:

“*I can imagine a future with increased confidence, hopefully, more engagement with family and friends, increased energy levels, and less negative self-talk.*”

Participant 586 described a similar desire for feeling comfortable with social engagement:

“*I feel like I’d be more comfortable in a lot of work or social settings, not always feeling like the largest person in the room or being self-conscious about what I’m wearing and people looking at me and ‘oh my God do I really look like that on camera’ sort of thing.*”

Participants could visualise a future for themselves where they possess increased confidence, increased energy, and a positive view of self. Achieving control over their eating was perceived as their route to a more enjoyable social life.

## 4. Discussion

This study builds on previous research that identified control and compulsion as factors that substantially affect eating behaviours, by exploring the concepts of control and the drivers of overeating (compulsion) in Australian adults with addictive eating behaviours [8]. Interviews with participants in the TRACE program were designed to capture insight into the roles that control and compulsion play in their eating behaviours. Specifically, the interviews examined how situations driving overeating, play in their eating behaviours.

Two main themes were constructed from the analysis: The paradox of addictive eating, and improvement of self. The paradox of addictive eating theme is consistent with wider research in substance use and behavioural addiction, in which paradoxical ideas and behaviours are common [23,24]. A prominent paradox in those with addictive eating in the current study is the love/hate relationship with food and eating. What is unknown is the inner complexities of these feelings. One possibility is that individuals with addictive eating behaviours blame themselves for their love of food driving their addictive eating, rather than considering that their behaviour may be related to non-controllable factors, such as mental health or behaviors within their control (such as specific thought patterns). In the current context, an appropriate definition for ‘love’ would encompass actively desiring or taking pleasure in food or eating [25]. However, participants describe love of food as a negative drive which evolves into a hatred toward eating. The love and hate relationship with food is a preoccupation, yet, eating is a familiar enemy, a distractor to be relied on when they need it. Given this apparent love/hate paradox, our findings add to the discussion that food or eating may have addictive attributes.

In a study undertaken by Dennis (2017) [23], the love/hate experienced in those who inject drugs, is expressed as a sense of pleasure when it should not be seen as a pleasurable undertaking. This idea resonates with how the participants in the current study express their perceptions of the drivers of overeating. They seek pleasure in food to assist with easing stress and to bring comfort, whereas the eating often produces a non-pleasurable effect. This similar paradox is also applicable to the pursuit of happiness by the participants. Many participants linked gaining control of their eating behaviours to increased happiness. Interestingly, participants always expressed that being in control of their eating behaviours would lead to happiness, not the alternative, that increasing happiness would change their eating behaviours. Through this expression, it is perceived that participants are valuing happiness, and achieving higher levels of happiness, as a clear goal in their lives. Interestingly, research has shown those who highly value happiness are more likely to experience unhappiness, which again, presents a paradox in the experience of individuals with addictive eating behaviours [26,27].

The boredom and busyness dichotomy adds another layer to the addictive eating paradox. Participants reported eating out of boredom, but also acknowledged overeating when feeling overwhelmed by tasks or responsibilities. While keeping busy was seen as a strategy to avoid eating, it paradoxically triggered a desire to search for and consume food. This interplay between boredom and busyness highlights the contradictory behaviours associated with addictive eating. A recent study on boredom identifies that boredom can manifest when an individual perceives a situation or task as unimportant and highlights that feeling overwhelmed, e.g., too busy, can lead to a loss of attention [28]. Thus, boredom and busyness can be recognised as similar states of attention. Considering the relationship between boredom and busyness, coping strategies for behaviour management during demanding periods may be warranted alongside boredom strategies [28,29].

The large amount of time participants spend thinking about, and acquiring food, coupled with the investment of time into their beliefs and perceptions of “normal” appears to contribute a substantial quantity of mental capacity, and aligns with symptom three in the YFAS 2.0 (Much time/activity to obtain, use, recover). The time and emotional resources participants expend thinking about how they are perceived by others could be diverted into strengthening productive coping strategies.

There are parallels between the time and coping strategies used by individuals with addictive eating compared to other addictive behaviours. Just as the participants in the current study expressed struggles and the need for positive coping strategies, individuals in addiction management programs face similar challenges, including emotion regulation [30]. A study into the use of gaming as a coping strategy could be a useful model to refer to when considering addictive eating [31]. The review proposed that removing gaming is not effective in the management of the addictive behaviour, but instead education around the motivations for the behaviour and the implementation of alternative tools to manage stress is highly beneficial [31].

The concept of ‘normality’ can often lead to self-stigmatisation and ‘othering’ behaviour [32]. ‘Othering’ has been a common discourse in historical discussions around substance-use and addictive behaviours which can reduce individuals to being viewed as unworthy compared to ‘normal’ individuals [33]. ‘Othering’ in terms of addictive behaviour holds the individual responsible for their behaviour and stereotypes them as a particular type of person [34]. Those with addictive eating behaviours appear to have internalised these similar beliefs and place the blame on themselves for not being ‘normal’ and adhering to societal expectations around eating habits. However, it is essential to bring into question what defines “normal” when it comes to eating behaviour. A study looking into the motivations for “normal” eating found many complex motives including individually perceived social norms, biological variables, and avoidance mechanisms driven by weight concerns [35]. It is important to consider that everyone faces challenges when it comes to their relationship with food, regardless of the perceived “normality”. Understanding how individuals who have worked to improve their relationship with food have achieved this can provide valuable insights. This may include increasing levels of self-compassion and strategies to improve interpersonal relationships [36,37]. These experiences of transformation may provide an inclusive understanding of the navigation of moving from ‘other’ to ‘normal’.

This ‘othering’ by those with addictive eating behaviours also leads to a lack of social confidence and the giving up of social situations. This aligns with the YFAS 2.0 symptoms of “Important social, occupational, or recreational activities given up or reduced” and “Continue use despite social or interpersonal problems” [2]. Considering this, future management programs could incorporate challenging these thoughts of social exclusion and provide strategies for individuals to normalise the process of eating and being in social situations where they may feel uncomfortable around the food on offer, or uncomfortable with their body size or shape. Interestingly, the findings in the current study appear to be in contrast with the findings in the qualitative study of the YFAS 2.0 by Schiestl et al. (2022) [14]. Schiestl et al. reported that the problem-focused symptom of giving up activities was rarely endorsed by the participants in their study, whereas in the current study, it was discussed by participants frequently in the context of avoiding social interactions due to their eating or how they felt others would perceive them in social situations [14].

Alongside the self-stigmatising in the context of ‘normality’ is the internalised weight stigma experienced by adults with addictive eating behaviours [32]. Societal pressures around body image, body shape, and the stereotyping of eating behaviours can lead to the internalisation of shame and guilt. Contributing to these feelings of shame and guilt may be the prominent messaging delivered to the public of the correlation between health outcomes and BMI which is not always supported in the research [38]. For those with addictive eating behaviours, this internalised weight stigma can intensify these feelings of self-blame and low self-esteem, making it difficult to implement effective behaviour change strategies, indicating a need for psychoeducation components to interventions that target addictive eating [39]. An emerging area of research in resistance to weight stigma may be a useful framework for inclusion in management programs for addictive eating behaviours [40]. Meadows and Higgs (2022) [40] utilize the social identity theory framework to challenge individuals’ beliefs and suggest that challenging the inequitable treatment of those not matching the expected social norms may increase resistance to internalised weight stigma. It is noted, however, that this resistance to internalised stigma has increased effectiveness when deployed in a group setting, which should be a consideration for future management programs. In turn, the social identity theory framework could be implemented to challenge the perceived ‘normality’ around controlling eating behaviours to assist adults with addictive eating to resist the internalisation of shame and guilt in terms of their food consumption.

The fear of missing out (FoMO) on foods and eating experiences was a prominent concept for the participants. This idea of FoMO, which similarly to addictive eating has associations with depression, anxiety, and boredom, has been explored in other areas including social media use and alcohol use [41,42,43,44,45]. However, most of this research has been conducted in adolescents and young adults. A similar interpretation of FoMO can be applied to food in that one may feel they are missing out on an important experience if they do not partake in eating a food they enjoy or a food they have not tasted before. This idea of FoMO may be exacerbated in those with addictive eating when confronted with marketing of new foods, or new food combinations. This may bring to the forefront the large impact that the current food environments and marketing of highly processed foods may have on individuals struggling with addictive eating behaviours who also experience feelings of FoMO.

### 4.1. Practical Implications

The insights gained from the current research have practical implications for the effective management of addictive eating. By understanding more about compulsion and control from individuals with addictive eating, we can further clarify the similarities and differences between addictive-like eating and eating disorders including AN, BED, and BN. In managing addictive eating, incorporating techniques such as cognitive therapy may prove beneficial. For example, challenging individual’s beliefs about control through constructive questioning may help them to examine the accuracy and the value they place on their expectations relating to food and eating behaviours. Rational disputation of eating expectations and beliefs with management strategies that include coping skills may assist in reshaping individual perspective and promoting positive behaviour change.

Furthermore, future interventions should consider the psychological constructs sought by individuals with addictive eating behaviours, such as happiness. Strategies for improved emotional regulation may play a pivotal role in enhancing overall happiness and health outcomes. By targeting emotional regulation, these programs can better equip individuals with skills to cope with behavioural cues and, in turn, reduce the dependence on food as a means of ineffective self-regulation.

### 4.2. Strengths and Limitations

This study provides a robust insight into the concepts of control and compulsion in the context of addictive eating behaviours. The main strength of this study was the capture of the experiences of Australian adults with addictive eating behaviours who were actively thinking about their addictive eating behaviours due to being enrolled in the TRACE program. Another strength was the high levels of engagement of reflexivity by the researchers to develop the themes and ensure accurate interpretation and presentation of the themes within the analysis. The study was limited due to the time constraints within the program, which may have reduced the opportunity to explore interesting ideas further. The results may not be transferrable to all populations due to the gender imbalance of participants, with only six participants being men; the study did not account for non-binary participants. Aboriginal and Torres Strait Islander demographic information was collected at baseline; however, there were no participants from this population group in the current study. No other ethnicity data was collected, which is an additional limitation. There is also a lack of representation from those with a BMI less than 25 kg/m^2^. Food access in the context of food insecurity was not addressed in the current study and should be a consideration for future research in addictive eating. It should also be noted that part of the study occurred during the COVID-19 lockdowns in Australia and, therefore, some of the concerns highlighted could have been exacerbated in the participants’ lives at the time of the interviews.

## 5. Conclusions

This study expands on the previously researched notions of compulsion and control in the context of addictive eating. Examining the participants’ perspectives on the phenomena of compulsion and control through the TRACE program has provided valuable insights regarding the paradoxical nature of addictive eating, aligning with multiple other substance-use disorders and addictive disorders. Multiple paradoxes were constructed from participant responses, including the love/hate relationship with food, the drivers of overeating, and the interplay between boredom and busyness. Happiness was strongly linked to control by the participants, highlighting the importance of emotional regulation in managing addictive eating behaviours. This study highlights the inordinate amount of time and mental capacity invested in thoughts about food and eating, and the need to incorporate psychoeducation into the management of addictive eating to challenge the internalised thoughts around weight status and notions of ‘normal’ eating. These findings emphasise the need to provide support and strategies to challenge negative, internalised thoughts. Overall, this research expands the understanding of addictive eating behaviours and presents new ideas to contribute to the effectiveness of interventions for individuals struggling with these complex eating issues.

## Figures and Tables

**Table 1:** Demographics of TRACE participants who completed the qualitative sub-study.

	Total Sample (*n* = 40)
	Mean ± SD (range) or *n* (%)
Age (years)	47.7 ± 13.3 (23.0–74.0)
Sex	
Female	34 (85.0)
BMI (kg/m^2^)	36.2 ± 6.0 (25.1–48.1)
Highest qualification	
School certificate/Higher school certificate (Year 10/Year 12 or equivalent)	8 (20.0)
Trade or diploma	8 (20.0)
Undergraduate university degree (e.g., Bachelor)	11 (27.5)
Postgraduate university degree (e.g., Graduate Certificate or Masters) or Higher research degree (e.g., PhD)	13 (22.5)
Employment status	
Employed—Full time	15 (37.5)
Employed—Part time	9 (22.5)
Employed—Casual	5 (12.5)
Student	3 (7.5)
Unemployed—looking for work	2 (5.0)
Unemployed—not looking for work	6 (15.0)
Marital status	
In a relationship, not married	6 (15.0)
Married or in a domestic partnership	28 (70.0)
Divorced, separated, or widowed	6 (15.0)
Current living situation	
Renting	11 (27.5)
Own home/Family home	28 (70.0)
Other	1 (2.5)
Currently living with	
Partner	31 (77.5)
Children	20 (50.0)
Flatmates or friends	2 (5.0)
Alone	5 (12.5)

**Table 2:** Themes and sub-themes related to compulsion and control.

Themes	Subthemes
The addictive eating paradox	The love of food Drivers of overeating The boredom and busyness dichotomy
2. Striving to transition from ‘other’ to ‘normal’	Mending psychological and health impacts Desiring normality Social Confidence

## Data Availability

Authors can be contacted regarding access to datasets related to the current study.

## References

[1] American Psychiatric Association (2022). Diagnostic and Statistical Manual of Mental Disorders.

[2] Gearhardt A.N., Corbin W.R., Brownell K.D. (2016). Development of the Yale Food Addiction Scale Version 2.0. Psychol. Addict. Behav..

[3] Gordon E.L., Ariel-Donges A.H., Bauman V., Merlo L.J. (2018). What Is the Evidence for “Food Addiction?” A Systematic Review. Nutrients.

[4] Gearhardt A.N., Hebebrand J. (2021). The concept of “food addiction” helps inform the understanding of overeating and obesity: Debate Consensus. Am. J. Clin. Nutr..

[5] Finlayson G. (2017). Food addiction and obesity: Unnecessary medicalization of hedonic overeating. Nat. Rev. Endocrinol..

[6] Hebebrand J., Albayrak Ö., Adan R., Antel J., Dieguez C., de Jong J., Leng G., Menzies J., Mercer J.G., Murphy M. (2014). “Eating addiction”, rather than “food addiction”, better captures addictive-like eating behavior. Neurosci. Biobehav. Rev..

[7] Leary M., Pursey K.M., Verdejo-Garcia A., Burrows T.L. (2021). Current Intervention Treatments for Food Addiction: A Systematic Review. Behav. Sci..

[8] Collins R., Haracz K., Leary M., Rollo M., Burrows T. (2020). No control and overwhelming cravings: Australian adults’ perspectives on the experience of food addiction. Appetite.

[9] Lacroix E., Oliveira E., de Castro J.S., Cabral J.R., Tavares H., von Ranson K.M. (2019). “There is no way to avoid the first bite”: A qualitative investigation of addictive-like eating in treatment-seeking Brazilian women and men. Appetite.

[10] Lacroix E., von Ranson K.M. (2020). Lived Experience and Defining Addictive-Like Eating: A Synthesis of Qualitative Research. Curr. Addict. Rep..

[11] PPaterson C., Lacroix E., von Ranson K.M. (2019). Conceptualizing addictive-like eating: A qualitative analysis. Appetite.

[12] Burrows T., Kay-Lambkin F., Pursey K., Skinner J., Dayas C. (2018). Food addiction and associations with mental health symptoms: A systematic review with meta-analysis. J. Hum. Nutr. Diet..

[13] Samela T., Innamorati M., Lester D., Raimondi G., Giupponi G., Imperatori C., Contardi A., Fabbricatore M. (2021). The association between adult ADHD and food addiction: A mediation analysis. Appetite.

[14] Schiestl E.T., Wolfson J.A., Gearhardt A.N. (2022). The qualitative evaluation of the Yale Food addiction scale 2.0. Appetite.

[15] Luigjes J., Lorenzetti V., de Haan S., Youssef G.J., Murawski C., Sjoerds Z., Brink W.v.D., Denys D., Fontenelle L.F., Yücel M. (2019). Defining Compulsive Behavior. Neuropsychol. Rev..

[16] Song S., Zilverstand A., Gui W., Pan X., Zhou X. (2022). Reducing craving and consumption in individuals with drug addiction, obesity or overeating through neuromodulation intervention: A systematic review and meta-analysis of its follow-up effects. Addiction.

[17] Whatnall M., Skinner J.A., Leary M., Burrows T.L. (2022). Food Addiction: A Deep Dive into ‘Loss of Control’ and ‘Craving’. Curr. Addict. Rep..

[18] Meule A., Westenhöfer J., Kübler A. (2011). Food cravings mediate the relationship between rigid, but not flexible control of eating behavior and dieting success. Appetite.

[19] Braun V., Clarke V. (2006). Using thematic analysis in psychology. Qual. Res. Psychol..

[20] Skinner J., Whatnall M., Leary M., Collins R., Pursey K.M., Verdejo-García A., Hay P.J., Baker A.L., Hides L., Paxton S.J. (2023). Examining the efficacy of a telehealth intervention targeting addictive eating in Australian adults (the TRACE Programme): A randomised controlled trial protocol. BMJ Open.

[21] Prnjak K., Mitchison D., Griffiths S., Mond J., Gideon N., Serpell L., Hay P. (2020). Further development of the 12-item EDE-QS: Identifying a cut-off for screening purposes. BMC Psychiatry.

[22] QSR International NVivo Product Editions. https://support.qsrinternational.com/nvivo/s/article/NV12Win-NVivo-Product-editions.

[23] Dennis F. (2017). Conceiving of addicted pleasures: A ‘modern’ paradox. Int. J. Drug Policy.

[24] Vandermause R.K. (2012). Being wholesome: The paradox of methamphetamine addiction and recovery—A hermeneutical phenomenological interpretation within an interdisciplinary, transmethodological study. Qual. Soc. Work..

[25] (2023). Merriam-Webster. https://www.merriam-webster.com/.

[26] Mauss I.B., Tamir M., Anderson C.L., Savino N.S. (2011). Can seeking happiness make people unhappy? Paradoxical effects of valuing happiness. Emotion.

[27] Zerwas F.K., Ford B.Q. (2021). The paradox of pursuing happiness. Curr. Opin. Behav. Sci..

[28] Westgate E.C. (2019). Why Boredom Is Interesting. Curr. Dir. Psychol. Sci..

[29] Westgate E.C., Wilson T.D., Gilbert D.T. (2017). With a little help for our thoughts: Making it easier to think for pleasure. Emotion.

[30] Extremera N., Quintana-Orts C., Sánchez-Álvarez N., Rey L. (2019). The Role of Cognitive Emotion Regulation Strategies on Problematic Smartphone Use: Comparison between Problematic and Non-Problematic Adolescent Users. Int. J. Environ. Res. Public Health.

[31] Melodia F., Canale N., Griffiths M.D. (2022). The Role of Avoidance Coping and Escape Motives in Problematic Online Gaming: A Systematic Literature Review. Int. J. Ment. Health Addict..

[32] Meadows A., Higgs S. (2020). Internalized weight stigma and the progression of food addiction over time. Body Image.

[33] Atkinson A.M., Sumnall H. (2020). Neo-liberal discourse of substance use in the UK reality TV show, *The Jeremy Kyle Show*. Drugs Educ. Prev. Policy.

[34] Morris J., Melia C. (2019). Challenging the language of alcohol problems. Psychologist.

[35] Renner B., Sproesser G., Strohbach S., Schupp H.T. (2012). Why we eat what we eat. The Eating Motivation Survey (TEMS). Appetite.

[36] Carbonneau N., Holding A., Lavigne G., Robitaille J. (2021). Feel Good, Eat Better: The Role of Self-Compassion and Body Esteem in Mothers’ Healthy Eating Behaviours. Nutrients.

[37] Pettersen G., Rosenvinge J.H. (2002). Improvement and Recovery from Eating Disorders: A Patient Perspective. Eat. Disord..

[38] Tomiyama A.J., Hunger J.M., Nguyen-Cuu J., Wells C. (2016). Misclassification of cardiometabolic health when using body mass index categories in NHANES 2005–2012. Int. J. Obes..

[39] Niveau N., New B., Beaudoin M. (2021). Self-esteem Interventions in Adults—A Systematic Review and Meta-analysis. J. Res. Pers..

[40] Meadows A., Higgs S. (2022). Challenging oppression: A social identity model of stigma resistance in higher-weight individuals. Body Image.

[41] Alutaybi A., Al-Thani D., McAlaney J., Ali R. (2020). Combating Fear of Missing Out (FoMO) on Social Media: The FoMO-R Method. Int. J. Environ. Res. Public Health.

[42] Gupta M., Sharma A. (2021). Fear of missing out: A brief overview of origin, theoretical underpinnings and relationship with mental health. World J. Clin. Cases.

[43] Riordan B.C., Flett J.A.M., Cody L.M., Conner T.S., Scarf D. (2021). The Fear of Missing Out (FoMO) and event-specific drinking: The relationship between FoMO and alcohol use, harm, and breath alcohol concentration during orientation week. Curr. Psychol..

[44] Liu X., Liu T., Zhou Z., Wan F. (2023). The effect of fear of missing out on mental health: Differences in different solitude behaviors. BMC Psychol..

[45] Wolniewicz C.A., Rozgonjuk D., Elhai J.D. (2020). Boredom proneness and fear of missing out mediate relations between depression and anxiety with problematic smartphone use. Hum. Behav. Emerg. Technol..

